# Artificial Intelligence in Endodontic Education: A Systematic Review with Frequentist and Bayesian Meta-Analysis of Student-Based Evidence

**DOI:** 10.3390/dj13110489

**Published:** 2025-10-23

**Authors:** Carlos M. Ardila, Eliana Pineda-Vélez, Anny M. Vivares-Builes

**Affiliations:** 1 Department of Periodontics, Saveetha Dental College, and Hospitals, Saveetha Institute of Medical and Technical Sciences, Saveetha University, Saveetha 600077, India; 2 Biomedical Stomatology Research Group, Basic Sciences Department, Faculty of Dentistry, Universidad de Antioquia U de A, Medellín 050010, Colombia; eliana.pineda@uam.edu.co (E.P.-V.); anny.vivares@uam.edu.co (A.M.V.-B.); 3 Faculty of Dentistry, Institución Universitaria Visión de las Américas, Medellín 050040, Colombia

**Keywords:** endodontic education, artificial intelligence, dental students, large language models, convolutional neural networks, diagnostic accuracy, meta-analysis, Bayesian meta-analysis, educational assessment

## Abstract

Background/Objectives: Artificial intelligence (AI) is entering dental curricula, yet its educational value in endodontics remains unclear. This review synthesized student-based evidence on AI in endodontics, primarily comparing AI vs. students on diagnostic tasks as an educational endpoint and secondarily considering assessment tasks relevant to training. Methods: PubMed/MEDLINE, Embase, Scopus, and Web of Science were searched in July 2025. Eligible studies involved dental students using AI in endodontic tasks or applied AI to student-generated outputs. For diagnostic comparisons we performed random-effects meta-analysis and a complementary Bayesian random-effects model with weakly informative priors. Risk of bias used QUADAS-2; certainty used GRADE. Results: Five studies met inclusion. Two provided complete mean–SD data for the primary meta-analysis and one contributed to a sensitivity model after SD imputation; two were summarized narratively (AUC/F1 only). Pooled effects favored AI: Hedges g = 1.48 (95% CI 0.60–2.36; I^2^ ≈ 84%); sensitivity (k = 3) g = 1.45 (95% CI 0.77–2.14; I^2^ ≈ 77%). Across the two LLM studies with analyzable means/SDs, the pooled mean difference in accuracy was approximately +20 percentage points (AI − students). Bayesian analyses yielded posterior means near 1.5 with 95% credible intervals excluding 0 and P (*μ* > 0) ≈ 1.00. Educational outcomes were sparsely and non-standardly reported. Conclusions: Student-based evidence indicates that AI likely outperforms dental students on endodontic diagnostic tasks, supporting its use as an adjunct for formative tutoring, objective feedback, and more consistent assessment.

## 1. Introduction

Artificial intelligence (AI) is increasingly reshaping health education and clinical practice, and its emergence in dental education has opened new avenues for innovation [1,2,3]. AI systems, including convolutional neural networks (CNNs) and large language models (LLMs), have demonstrated the ability to perform image interpretation, pattern recognition, and complex decision-making tasks with high accuracy [4,5,6]. In dental academia, these tools are being harnessed not only to enhance diagnostic processes but also to support student learning and automate assessment workflows [7,8,9]. This shift has been catalyzed by technological advancements and the increasing demand for scalable, objective, and consistent educational methodologies [6,8].

In endodontics, diagnostic acumen is critical for determining treatment strategies and ensuring optimal clinical outcomes. Traditionally, dental students develop these competencies through lectures, supervised clinical training, and exposure to case scenarios. However, disparities in teaching quality, variability in clinical case complexity, and limited opportunities for repeated practice can hinder uniform skill acquisition. AI has therefore emerged as a promising adjunct in educational settings, capable of offering real-time feedback, individualized learning pathways, and automated performance evaluations [9,10,11,12]. In endodontic education, diagnostic tasks—such as pulp vitality testing and periapical status interpretation—are fundamental learning milestones used to evaluate students’ clinical reasoning and decision-making accuracy. At the undergraduate level, curricula explicitly require mastery of working-length determination and treat it as a routinely examined cornerstone skill. These competencies are particularly important in endodontics, where radiographic interpretation plays a central role—a domain in which CNNs have shown strong potential [13,14,15].

The diagnostic and analytical capacity of CNNs has been widely demonstrated across healthcare domains, facilitating classification, anomaly detection, and standardized measurement [4,5,6]. In dental education, these networks can contribute to consistent grading, reduction in examiner variability, and enhancement of student feedback loops [4,7,10]. Their integration also supports image-based learning strategies, particularly useful for strengthening anatomical and procedural understanding [7,13].

Alongside image-based AI, LLMs have emerged as valuable educational tools. These models can process large volumes of unstructured textual data, generate clinically relevant suggestions, and simulate patient interactions, thereby supporting clinical reasoning and assessment training [9,10]. They also enable automated formative feedback, helping to identify learning gaps and promote self-directed learning [9,11]. Incorporating these models into curricula aligns with the growing emphasis on digital literacy and decision-support tools in health education [1,2,3,7].

Despite this promise, evidence on the educational role of AI in endodontics remains fragmented. While several studies have evaluated AI applications in diagnostic and educational tasks, broader aspects such as standardized assessment, academic integrity verification, and automated feedback remain underexplored. Furthermore, little is known about student engagement, motivation, accessibility, and trust in AI-assisted learning, which limits understanding of how these technologies can be effectively integrated into curricula or evaluated through measurable educational proxies such as diagnostic accuracy and consistency in student performance.

Because few studies have directly measured learning or competency gains, diagnostic performance serves as a practical educational proxy reflecting students’ knowledge application, reasoning accuracy, and assessment reliability when supported by AI.

Accordingly, this systematic review and meta-analysis aimed to synthesize student-based evidence on the educational use of artificial intelligence in endodontics. Within this framework, we compared the diagnostic performance of AI systems and dental students as a measurable educational endpoint reflecting knowledge application and assessment accuracy. In an education-focused scope, we also considered key assessment tasks relevant to training as complementary evidence. We also summarized student-centered outcomes—such as engagement, motivation, and perceived usefulness—when reported.

## 2. Materials and Methods

### 2.1. Protocol and Registration

This systematic review and meta-analysis adhered to the Preferred Reporting Items for Systematic Reviews and Meta-Analyses (PRISMA) 2020 guidelines [16]; the completed PRISMA 2020 checklist is provided in the Supplementary Materials (Table S1). The protocol was prospectively registered in the International Prospective Register of Systematic Reviews (PROSPERO database CRD420251107002).

### 2.2. Eligibility Criteria

Studies were eligible if they involved dental students at any stage of formal training in endodontics (preclinical undergraduate, clinical undergraduate, postgraduate, or residency) and evaluated the application of AI models—including CNNs, LLMs, or hybrid tools—for teaching, assessment, feedback, or diagnostic support within an educational context. Eligible studies had to assess AI systems used directly by students or applied to student-generated data/deliverables (cases, images, radiographs, procedural outputs, or assessments produced during formal educational activities such as preclinical laboratory work, clinical training, or structured examinations).

The primary focus of inclusion was on studies exploring AI-assisted educational interventions in endodontics, such as integration into teaching, assessment, or academic integrity verification. Only studies with direct student participation were eligible; benchmarking studies based exclusively on expert comparators or indirect evaluations without student involvement were excluded.

Comparators were required to include student performance obtained within a defined educational or assessment framework using validated instruments. Study designs encompassed diagnostic accuracy studies, cross-sectional studies, validation studies, and randomized controlled trials.

Studies were excluded if they (i) focused exclusively on expert-level performance without any involvement of students or student-generated outputs, (ii) used only patient-derived clinical data without student participation or implementation in an educational setting, even if authors speculated on potential educational utility, (iii) assessed non-endodontic specialties without reporting separate endodontic outcomes, (iv) did not report relevant primary or secondary outcomes as defined in this review, or (v) were reviews, editorials, or protocols lacking original data.

### 2.3. Operational Definition of AI-Assisted Tools

For the purposes of this review, AI-assisted tools were defined as artificial intelligence systems either (i) used directly by dental students during educational activities, or (ii) applied to outputs generated by students in structured training contexts (radiographs, case reports, test responses, or procedural work). This definition emphasizes the educational deployment of AI within endodontics. Datasets derived solely from patient care, without student participation or connection to an educational setting, were excluded.

### 2.4. PICO Framework

- Population (P): Dental students undergoing formal training in endodontics at any stage, including preclinical undergraduate courses, clinical undergraduate courses, postgraduate students, and residents in endodontic specialty programs. Studies evaluating AI performance on validated student assessment instruments used in endodontic education were also eligible.
- Intervention (I): Use of AI-based tools (CNNs, LLMs, chatbots) integrated into learning activities, diagnostic assistance, or assessment of students, or applied to outputs generated by students within an educational setting.
- Comparator (C): Student performance obtained within validated educational or assessment frameworks.
- Outcomes (O): Primary outcomes included diagnostic performance metrics (accuracy, sensitivity, specificity, F1-score, and AUC) when AI results were directly compared with student performance. Secondary outcomes encompassed educational indicators such as assessment consistency, feedback quality, academic integrity verification, and student learning-related measures.

This framework guided the selection, synthesis, and interpretation of eligible studies to ensure clinical relevance and methodological consistency.

### 2.5. Information Sources and Search Strategy

A comprehensive search was performed across PubMed/MEDLINE, Embase, Scopus, and Web of Science for studies published from database inception to July 2025. Supplementary searches were conducted in Google Scholar and through backward and forward citation chasing of all included articles, and this process was documented in the PRISMA flow diagram. The strategy combined Medical Subject Headings (MeSH) and free-text terms related to “artificial intelligence,” “machine learning,” “deep learning,” “chatbot,” “large language model,” “endodontics,” “students,” “education,” “training,” and “assessment,” using Boolean operators. The search was designed to capture studies investigating the educational application of AI in endodontics with direct student participation. The complete search strategies for each database, including syntax and field tags, are provided in Supplementary Table S2.

### 2.6. Study Selection

Two independent reviewers screened the titles and abstracts using Rayyan. Full texts of potentially relevant articles were retrieved and evaluated against the predefined inclusion criteria, with a primary focus on the educational use of AI in endodontics. Disagreements were resolved by discussion or consultation with a third reviewer. The selection process is documented in the PRISMA flow diagram. A detailed list of full-text excluded studies with specific reasons for exclusion is provided in Supplementary Table S3.

### 2.7. Data Extraction

Data were extracted using a pre-piloted standardized form. Extracted items included study characteristics (authors, year, country), population details (student level), AI model used, comparator(s), outcome measures, and key findings. Data extraction was independently performed by two reviewers, with discrepancies resolved through discussion. For outcomes such as student engagement, accessibility, motivation, and trust, a structured narrative synthesis framework was applied. Qualitative statements, self-reported perceptions, and accessibility-related notes were thematically grouped according to the predefined domains in the protocol, ensuring consistency despite heterogeneous reporting across studies.

### 2.8. Outcome Measures

The primary quantitative outcome was diagnostic performance when AI was directly compared with student performance (accuracy, sensitivity, specificity, F1-score, AUC). Secondary, student-centered outcomes (perceptions of usefulness or ease of use) were summarized narratively when available. Radiographic measurement/assessment tasks were not classified as diagnosis; they were synthesized narratively and included only in sensitivity analyses.

### 2.9. Data Synthesis and Meta-Analysis

Given the anticipated variability in constructs and measurement instruments, educational outcomes (assessment consistency, feedback, integrity, and student perceptions) were planned to be synthesized narratively. For studies directly comparing AI with students on diagnostic tasks, we planned a random-effects meta-analysis using the Paule–Mandel estimator for between-study variance (τ^2^) and Hartung–Knapp adjustments for confidence intervals to accommodate the expected small number of studies. The primary effect size was prespecified as the standardized mean difference (Hedges g), defined so that positive values indicate better AI performance. As a clinically interpretable complement, we planned to pool mean differences in percentage points (MD; AI − students) when means and standard deviations (SD) were available. When multiple student cohorts were reported within a study, they were prespecified to be combined into a single student group using standard formulas for pooled means and SDs. If any study reported proportions without SDs, we planned a sensitivity analysis imputing SDs via a binomial approximation based on the number of evaluated items and assigning conservative sample sizes to avoid over-weighting. Heterogeneity was planned to be quantified using I^2^, τ^2^, and Cochran’s Q, and a 95% prediction interval for the pooled effect was planned a priori. Small-study effects/publication bias were not planned to be assessed unless ≥10 studies contributed to the quantitative synthesis. Frequentist analyses were planned in R (version 4.3.3) using standard routines (e.g., metafor/meta). Only studies of endodontic diagnosis with analyzable mean–SD data were pooled in the primary meta-analysis.

To aid interpretation with few studies, we planned a Bayesian random-effects meta-analysis, yi∼Normal (*μ*,vi + τ2), with weakly informative priors *μ*∼N (0,1) and τ∼Half-Normal (0.5). We planned to report the posterior mean of *μ*, its 95% credible interval, and the probabilities *P* (*μ* > 0), *P* (*μ* > 0.5), and *P* (*μ* > 1.0) (in SD units) as measures of probability of superiority. This Bayesian analysis was specified as a priori as a confirmatory/interpretive complement and did not alter study inclusion or exclusion criteria. Bayesian computations were planned in R version 4.3.3 using the bayesmeta package (version 2.6.0); posterior summaries and plots used posterior (1.5.0) and bayesplot (1.11.1).

### 2.10. Risk of Bias and Evidence Certainty

Risk of bias was assessed using the QUADAS-2 tool for diagnostic accuracy studies [17] and the Joanna Briggs Institute (JBI) checklist for cross-sectional studies [18]. Two reviewers performed assessments independently. Certainty of evidence was evaluated with the GRADE approach, considering risk of bias, inconsistency, indirectness, imprecision, and publication bias [19].

## 3. Results

### 3.1. Study Selection

**Figure 1:**
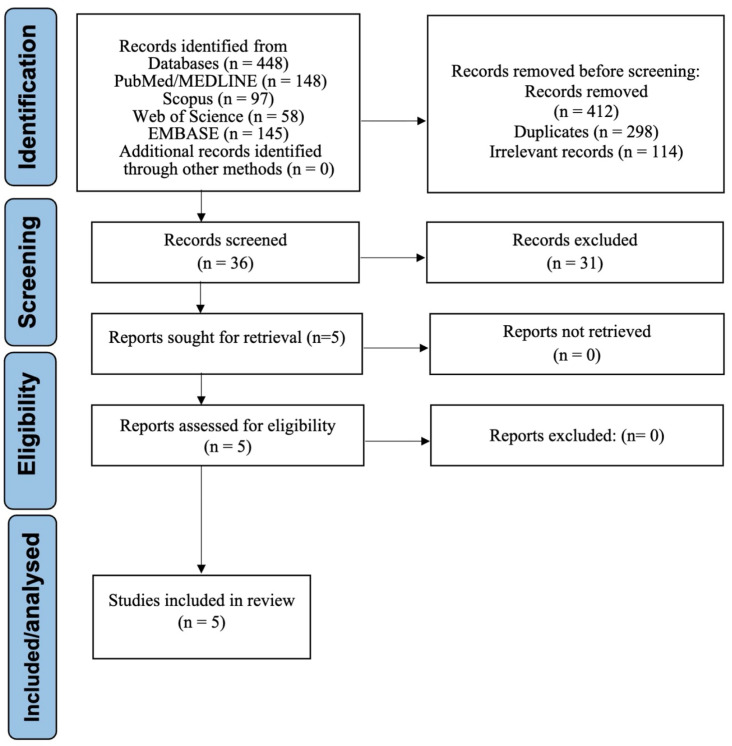
PRISMA flow diagram illustrating the selection process for studies included in the systematic review.

A total of 448 records were identified through database searches. Prior to screening, 412 records were excluded due to duplication, non-relevance based on title/abstract, or not meeting the topic scope (not focused on education, endodontics, or AI). This left 36 full-text articles assessed for eligibility. Of these, 31 studies were excluded for specific reasons, including absence of student participation or educational context, non-endodontic focus, insufficient reporting of AI-related outcomes, or editorials/protocols. Five studies met the inclusion criteria and were included in the qualitative synthesis [20,21,22,23,24]. Of these, two provided complete mean–SD data for inclusion in the primary random-effects meta-analysis [20,23]; one additional study contributed to a sensitivity meta-analysis after pre-specified SD imputation [21]; and two were synthesized narratively due to incompatible summary statistics [22,24]. The selection process is depicted in Figure 1.

### 3.2. Study Characteristics

The five included studies covered diagnostic tasks commonly taught in endodontics. Two studies evaluated LLMs on clinical/virtual case diagnosis (ChatGPT-4/4o) [20,23], and three evaluated CNN-based models on radiographic tasks—working-length determination [21], C-shaped canal detection on panoramic radiographs [22], and pulp-exposure prediction from radiographs [24]. Participants included undergraduate (junior/senior; third/fifth year) and postgraduate students. For quantitative synthesis, two studies [20,23] provided complete mean–SD data suitable for the primary random-effects meta-analysis; one study [21] contributed to a sensitivity meta-analysis after pre-specified SD imputation; two studies [22,24] were summarized narratively due to incompatible summary statistics. Basavanna et al. [21] evaluated a deep-learning model for working-length estimation on periapical radiographs versus postgraduate students; this was classified as a radiographic assessment task rather than diagnosis and was not included in the primary diagnostic meta-analysis. Table 1 details study tasks, AI models, comparators, and outcome metrics.

### 3.3. Diagnostic Accuracy of AI vs. Students

**Figure 2:**
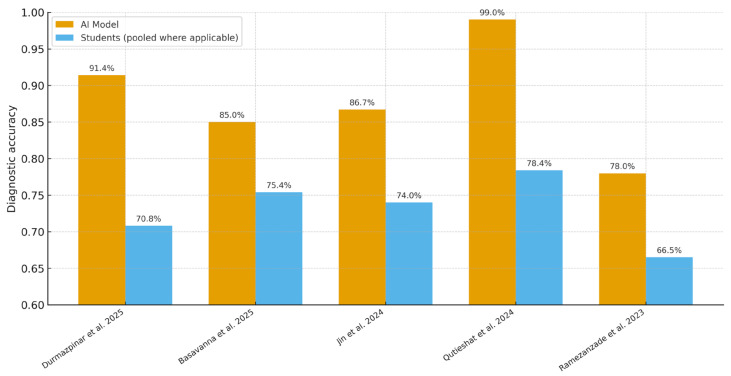
Diagnostic accuracy (%) of AI models versus dental students across the five included studies. When a study reported multiple student cohorts (e.g., 3rd/5th year; junior/senior), student values were pooled for visualization. In Ramezanzade [24], student accuracy is plotted as the midpoint of the reported 65–68% range. Comparative diagnostic accuracy of AI models versus dental students across included studies. Bars represent the mean diagnostic accuracy reported by each study: Durmazpinar et al. [20], Basavanna et al. [21], Jin et al. [22], Qutieshat et al. [23], and Ramezanzade et al. [24]. AI models consistently outperformed students, with the highest accuracy observed for Qutieshat et al. (99.0%).

All five included studies [20,21,22,23,24] directly compared AI models with dental students on endodontic diagnostic tasks (Figure 2). Across reported metrics (accuracy, AUC, F1), AI performance ranged from 71.0% to 99.0%, whereas student accuracy ranged from 60.8% to 79.7% (student F1 where reported: 58.0–61.0). For example, Qutieshat et al. [23] reported 99.0% accuracy for AI versus 79.7% (senior) and 77.0% (junior) students. Durmazpinar et al. [20] found 91.4% accuracy for ChatGPT-4o versus 79.5% (5th-year) and 60.8% (3rd-year) students. In radiographic tasks, Jin et al. [22] reported 86.7% AI accuracy (ResNet-101) with AUC 0.910 (95% CI 0.883–0.940); student accuracy varied by experience (74.0% graduate students; 77.2% novice dentists; 81.2% specialists). Basavanna et al. [21] showed 85.0% AI accuracy for working-length estimation versus 75.4% in postgraduate students (*p* = 0.0374). Ramezanzade et al. [24] reported F1 = 71.0 and accuracy = 78.0% for AI versus F1 = 58–61 and accuracy = 65–68% for students.

### 3.4. Educational Utility and Student Perceptions

Across the five included studies, no trial used validated instruments to measure student-centered outcomes (e.g., perceptions, engagement, motivation, accessibility, or trust), and no pre/post educational evaluations were reported. Two papers that compared AI with students on diagnostic tasks [20,23] included brief author comments on potential educational utility—for example, the opportunity for immediate feedback and exposure to varied case scenarios—but these statements were not accompanied by formal learner assessments or controlled educational designs. Consequently, no quantitative synthesis was conducted for this domain, and conclusions regarding educational utility remain exploratory and hypothesis-generating.

### 3.5. Student Engagement, Accessibility, Motivation, and Trust

Across the five included studies, no study administered validated instruments to evaluate student engagement, accessibility/usability of AI tools, motivation to learn, or trust in AI-assisted outputs. Likewise, no pre–post educational comparisons or structured qualitative methods (e.g., interviews/focus groups with formal coding) were reported. Two diagnostic studies [20,23] offered brief author observations about potential benefits (e.g., immediacy of feedback, ease of use), but these remarks were informal and unquantified. Given the paucity and heterogeneity of reporting, no quantitative pooling was undertaken; any statements regarding student-centered domains remain exploratory and are summarized narratively within the study descriptions.

### 3.6. AI-Augmented Learning Scenarios

None of the five included studies implemented an AI tool as a real-time, in-class or clinical feedback system, nor did they evaluate pre/post educational change attributable to AI. Two student-comparison studies with LLMs [20,23] briefly commented on the potential for immediate feedback and exposure to varied cases, but these remarks were not accompanied by formal educational measures. Radiographic CNN studies [22,24] reported diagnostic performance (AUC 0.910 in [22]; F1 = 0.71 in [24]) that could technically underpin feedback workflows, yet no study deployed such feedback during training. Accordingly, no quantitative synthesis was possible for AI-augmented learning scenarios, and conclusions remain exploratory.

### 3.7. Meta-Analysis of Diagnostic Performance

Of the five included studies, two provided complete mean–SD data for inclusion in the primary random-effects meta-analysis [20,23], and one [21] contributed to a sensitivity analysis with pre-specified SD imputation; two studies [22,24] reported diagnostic performance using AUC/F1 without SDs and were summarized narratively. The pooled effect favored AI over students on diagnostic tasks (Hedges g ≈ 1.5; Hartung–Knapp 95% CI ≈ 0.6–2.4; I^2^ ≈ 84%, τ^2^ ≈ 0.30; k = 2). A sensitivity model including [21] yielded a very similar pooled effect (g ≈ 1.45; HK 95% CI ≈ 0.77–2.14; I^2^ ≈ 77%, τ^2^ ≈ 0.32; k = 3). As pre-specified, the k = 3 sensitivity analysis includes the working-length assessment study; exclusion of this study does not change the direction of the effect. In absolute terms, both contributory studies showed an advantage of roughly +20 percentage points in accuracy for AI versus students.

Jin et al. [22] evaluated CNNs for C-shaped canal detection and reported AI accuracy 86.7% (ResNet-101) with AUC 0.910 (95% CI 0.883–0.940); student accuracy varied by experience (74.0% graduate; 77.2% novice dentists; 81.2% specialists). Ramezanzade et al. [24] reported AI F1 = 0.71 and accuracy = 78% versus students (F1 = 0.58–0.61; accuracy = 65–68%); these statistics were not pooled because they are not directly comparable to mean–SD accuracy and SDs were unavailable.

**Figure 3:**
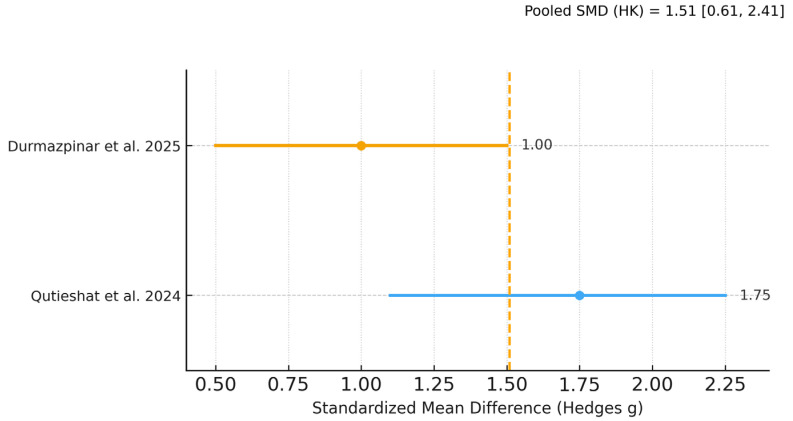
Random-effects meta-analysis (Paule–Mandel; Hartung–Knapp CIs) of AI versus student diagnostic performance for the two studies with mean–SD data (Durmazpinar et al. [20]; Qutieshat et al. [23]). Effect size is Hedges g (positive values favor AI). Student cohorts within a study were pooled.

**Figure 4:**
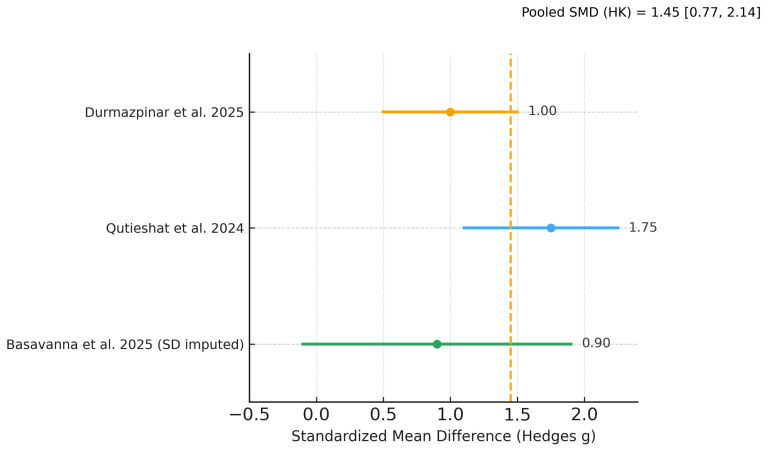
Sensitivity random-effects meta-analysis including Basavanna et al. [21] (2025) with pre-specified SD imputation (binomial approximation). Results are consistent in direction and magnitude with the primary analysis (Durmazpinar et al. [20]; Qutieshat et al. [23]; Basavanna et al. [21]). Effect size is Hedges g (positive values favor AI).

**Figure 5:**
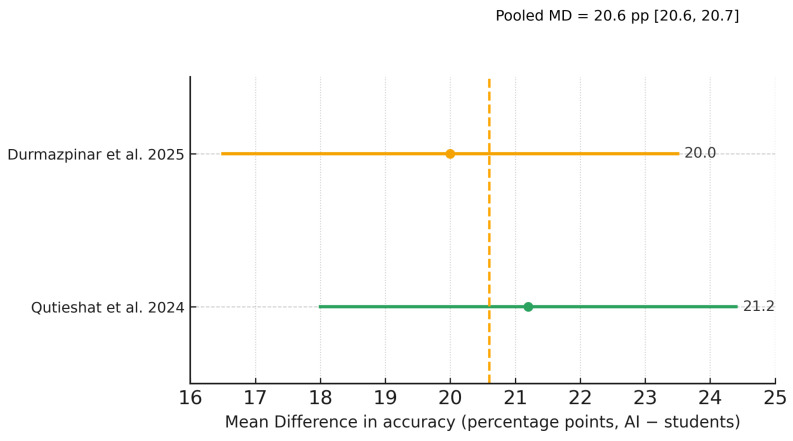
Random-effects meta-analysis of mean differences in diagnostic accuracy (percentage points, AI − students) for the two LLM studies with mean–SD data (Durmazpinar et al. [20]; Qutieshat et al. [23]). The dashed line marks the pooled mean difference (≈+20.6 pp). Student cohorts were pooled for analysis. The color of each point matches its corresponding confidence interval line.

Figure 3, Figure 4 and Figure 5 display the primary SMD meta-analysis, the sensitivity SMD meta-analysis, and the pooled mean difference (percentage points), respectively.

### 3.8. Exploratory Analyses

To complement the secondary meta-analysis and provide an intuitive metric, we computed unweighted percentage-point (pp) differences in accuracy (AI − students) for studies reporting accuracy on a comparable % scale [20,21,22,23,24] (Table 2). Using consistent student comparators (see Table 2 footnotes), the overall unweighted mean difference was +15.0 pp. By model class, the mean difference was +20.6 pp for LLMs and +11.3 pp for CNNs. A sensitivity analysis excluding Qutieshat et al. [23] (the largest observed effect) yielded +13.6 pp. As expected, Figure 5—which pools only the two LLM studies with analyzable mean–SD data [20,23]—aligns with the LLM subgroup estimate in Table 2 (≈+20.6 pp).

### 3.9. Bayesian Meta-Analysis

**Figure 6:**
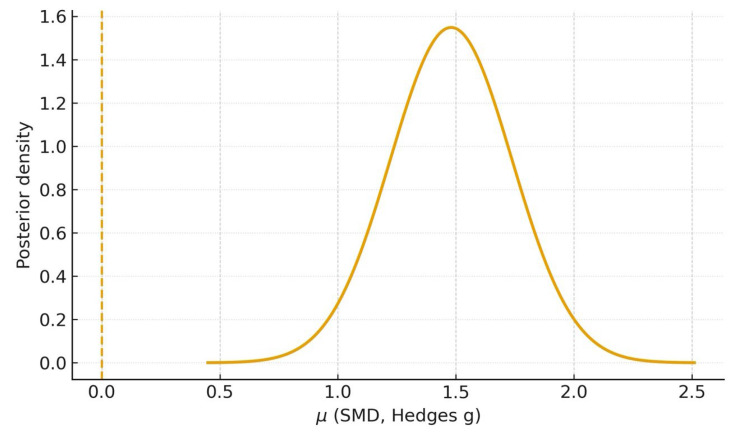
Posterior of *μ*—Primary analysis (k = 2). Bayesian random-effects posterior density for the overall standardized mean difference (Hedges g) using studies [20,23]. The dashed line marks *μ* = 0. Posterior mean 1.48; 95% CrI 0.98–1.99; P (*μ* > 0) ≈ 1.00; P (*μ* > 0.5) ≥ 0.99; P (*μ* > 1.0) ≈ 0.97.

**Figure 7:**
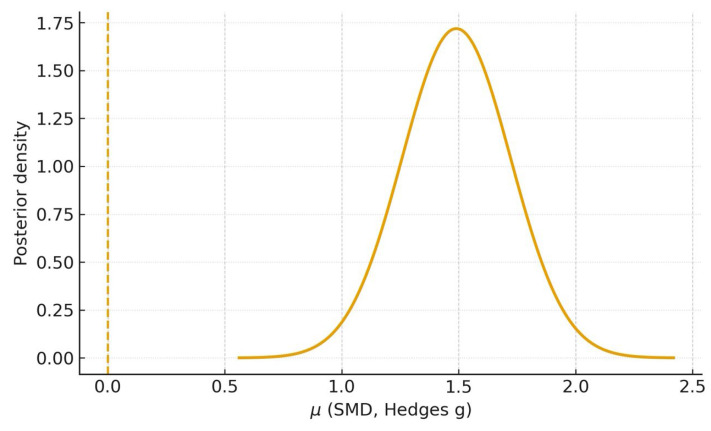
Posterior of *μ*—Sensitivity analysis (k = 3). Bayesian random-effects posterior density including Basavanna 2025 [21] with SD imputation. The dashed line marks *μ* = 0. Posterior mean 1.49; 95% CrI 1.05–1.96; P (*μ* > 0) ≈ 1.00; P (*μ* > 0.5) ≥ 0.99; P (*μ* > 1.0) ≈ 0.99.

Using the Bayesian random-effects model, analysis of the two studies with complete mean–SD data [20,23] yielded a posterior mean for the overall standardized mean difference of *μ* = 1.48 (95% CrI 0.98–1.99); probabilities of superiority were P (*μ* > 0) ≈ 1.00, P (*μ* > 0.5) ≥ 0.99, and P (*μ* > 1.0) ≈ 0.97, with a posterior median heterogeneity τ ≈ 0.24 (Figure 6). When the study with imputed SDs [21] was included, the posterior mean was *μ* = 1.49 (95% CrI 1.05–1.96), with P (*μ* > 0) ≈ 1.00, P (*μ* > 0.5) ≥ 0.99, P (*μ* > 1.0) ≈ 0.99, and τ ≈ 0.27 (Figure 7).

These Bayesian estimates corroborate the frequentist random-effects meta-analyses (Figure 3 and Figure 4) and, by providing direct probability statements, support a very high probability that AI outperforms students on the evaluated diagnostic tasks.

### 3.10. Risk of Bias Assessment

Risk of bias was assessed for all five included studies using the QUADAS-2 tool. Overall methodological quality was acceptable: three studies were at low risk [20,22,23] and two at moderate risk [21,24]. Domain-level judgements are presented in Table 3. Applicability concerns were low across studies; the moderate ratings were driven mainly by issues in participant selection, index-test conduct/reporting, and reference-standard/flow.

### 3.11. Certainty of Evidence (GRADE Assessment)

Using GRADE, the certainty of evidence for the comparative diagnostic outcome (AI vs. students) was judged moderate. For the primary meta-analysis [20,23], the pooled effect was Hedges g = 1.48 (95% CI 0.60–2.36) with substantial heterogeneity (I^2^ ≈ 84%). A sensitivity model adding the study with imputed SDs [21] gave a very similar estimate (g = 1.45; 95% CI 0.77–2.14; I^2^ ≈ 77%). We downgraded one level for inconsistency (high heterogeneity across tasks/models), did not downgrade for imprecision (intervals exclude the null and Bayesian CrIs are concordant), and did not downgrade for risk of bias or indirectness (three low-risk and two moderate-risk studies; tasks and populations are aligned with endodontic education). Although formal publication-bias testing was not performed (k < 10), we found no strong signals to warrant further downgrading. The consistently large effect size (SMD ≳ 1.0) across the five student-based studies supports upgrading one level for magnitude, yielding an overall judgment of moderate certainty that AI likely outperforms students on diagnostic accuracy as a complementary educational endpoint (Table 4).

## 4. Discussion

This systematic review and meta-analysis synthesized five student-based studies evaluating AI for endodontic diagnostic tasks [20,21,22,23,24]. Although diagnostic performance dominated the reported outcomes, this metric was interpreted within an educational framework—as an indicator of applied learning, decision-making competence, and potential formative assessment utility in endodontic training. Across these studies, AI models—both LLMs for case diagnosis and CNNs for radiograph-based tasks—consistently outperformed dental students. In the primary random-effects meta-analysis the pooled effect favored AI with Hedges g = 1.48 (95% CI 0.60–2.36; I^2^ ≈ 84%); a sensitivity model that included one study with pre-specified SD imputation gave a very similar estimate (g = 1.45; 95% CI 0.77–2.14; I^2^ ≈ 77%). On an absolute scale, both contributory LLM studies showed roughly +20 percentage-point higher accuracy for AI than for pooled student cohorts, which is consistent with the unweighted exploratory differences observed across all five studies (Table 2; Figure 3, Figure 4 and Figure 5).

The Bayesian random-effects analyses corroborated these findings, yielding posterior means of *μ* = 1.48 (95% CrI 0.98–1.99; k = 2) and *μ* = 1.49 (95% CrI 1.05–1.96; k = 3) with P (*μ* > 0) ≈ 1.00 and P (*μ* > 0.5) ≥ 0.99 (Figure 6 and Figure 7). These probability statements reinforce that, given the available student-based evidence, there is a very high probability that AI surpasses students on the evaluated diagnostic tasks.

These findings align with prior evidence showing that AI models often achieve superior diagnostic accuracy compared with dental students. For example, Qutieshat et al. [23] reported 99.0% accuracy for ChatGPT-4 in endodontic case diagnosis, markedly higher than senior (79.7%) and junior (77.0%) students. Likewise, Jin et al. [22] reported 86.7% accuracy for a ResNet-101 CNN with AUC 0.910 (95% CI 0.883–0.940); student accuracy varied by experience (74.0% graduate students, 77.2% novice dentists, 81.2% specialists). Other imaging reports also suggest that CNNs can match or exceed human raters for challenging anatomical variants [25]. While these results reinforce the robustness of AI on diagnostic tasks, they should be interpreted here as secondary evidence—useful to contextualize performance gaps rather than to quantify educational benefit.

Extending this pattern, Durmazpinar et al. [20] evaluated ChatGPT-4o on a set of clinical questions and observed 91.4% accuracy, exceeding both 3rd-year (60.8%) and 5th-year (79.5%) students. These results are concordant with work advocating multimodal AI systems—integrating textual and visual inputs—for educational diagnostics [26,27]. In practice, such systems could function as supplemental diagnostic tutors, offering immediate, case-specific rationales. Their integration into OSCE stations or case-based seminars may help translate theoretical knowledge into clinical decision-making, especially for early-stage learners. Nevertheless, as with other benchmarking comparisons, these outcomes remain indirect; their pedagogical value should be verified with learning endpoints (knowledge gain, retention, calibration of diagnostic thresholds, and learner trust).

In a procedural context, Basavanna et al. [21] showed that a deep-learning system reached 85.0% accuracy for working-length estimation, outperforming postgraduate students (75.4%, *p* = 0.0374). The study’s strength lies in its direct comparison with trained operators, an important step toward clinical credibility [28]. Educationally, similar tools could be deployed in preclinical simulation labs or as chairside decision supports to provide objective feedback and to standardize competency assessment in skill-based procedures. However, as elsewhere, the educational contribution is inferred rather than measured, underscoring the need for trials that pair performance metrics with learner outcomes.

Beyond comparative accuracy, AI has been explored for academic integrity in endodontic education. Ibrahim et al. [29] developed a Siamese neural-network framework to flag inconsistencies in student-generated radiographs from preclinical procedures, achieving 89.3% accuracy. Although student performance was not evaluated, the study illustrates the feasibility of auditing tools that detect procedural substitution or image manipulation—issues that have been reported in simulated dental assessments [30]. Incorporating verification systems of this type could enhance fairness, strengthen institutional policies, and reduce reliance on manual oversight.

Standardized assessment is another area of relevance. Ayhan et al. [31] proposed a YOLO-based system for automated evaluation of root-canal filling quality from student radiographs. The work highlights the subjectivity of faculty grading—well documented in prior literature [32,33]—and shows how expert criteria can be applied consistently to student outputs. While not eligible for our quantitative synthesis, such approaches point to AI as a mechanism to improve equity and reliability in educational assessment, complementing rather than replacing instructor judgment.

Importantly, higher diagnostic accuracy does not automatically translate into educational gains. Ramezanzade et al. [24]—who compared AI-based pulp-exposure prediction with student performance—observed limited immediate improvement when students were guided by the model. The authors emphasized explainability and trust as prerequisites for learning impact, a point echoed elsewhere [34]. Without transparent rationales and actionable feedback, AI tools risk offering “black-box answers” that students cannot internalize. These findings support embedding AI within pedagogical frameworks that prompt reflection and reasoning rather than positioning AI as a substitute for clinical judgment.

Accordingly, while many included studies contrasted AI with students on diagnostic tasks, this design serves an educational purpose: it reflects how AI can scaffold student reasoning in key diagnostic domains that are central to endodontic competence, including pulp and periapical diagnosis, radiographic interpretation, and working-length determination. Demonstrating pedagogical value requires validated, learner-centered outcomes—knowledge acquisition and retention, changes in diagnostic calibration, usability and trust scales, and effects on OSCE or clinical performance—ideally within prospective or randomized designs. Future work should therefore integrate these endpoints alongside diagnostic metrics, ensuring that AI is evaluated for its effectiveness as a teaching tool, not only for its algorithmic accuracy.

Translating these findings into actual learning outcomes requires anchoring diagnostic gains to recognized educational frameworks and measures [35,36]. In competency-based curricula and along Miller’s pyramid (“shows-how/does”), AI can function as a formative scaffold—driving deliberate practice, standardized feedback, and diagnostic calibration across varied cases. Emerging evidence shows that AI-generated individualized feedback can positively influence learner performance in medical education [37], and LLM-supported simulations can structure clinical reasoning and provide actionable feedback for improvement [38]. Accordingly, appropriate outcomes for future endodontic studies include OSCE-style diagnostic stations, rubric-based case reasoning, pre/post calibration in radiographic interpretation, and retention/transfer tests that reflect progression from “knows-how” to “shows-how/does” [39].

Beyond absolute effect sizes, several parameters likely modulated the observed AI–student differences. First, the task domain matters—case-level diagnosis versus single-label condition classification and radiographic assessment (e.g., working-length estimation) impose different cognitive/measurement demands that can benefit models optimized for pattern recognition [40]. Second, in LLM settings, prompt design and the requirement for stepwise rationales can alter stability and accuracy, whereas unstructured prompts may inflate variance [41]. Third, the choice of metrics under potential class imbalance (accuracy vs. AUC/F1) can change conclusions and comparability across studies [42]. Finally, validation strategy and transparent reporting (internal vs. external validation, data shifts, and model/version specification) critically affect generalizability and reproducibility and should be standardized in endodontic education research using AI [43].

While this review demonstrated that AI generally outperforms dental students in diagnostic accuracy, this level of comparison reflects formative rather than expert benchmarking. For responsible curricular integration, future educational applications should ensure that AI systems achieve or approximate expert-level diagnostic competence (e.g., endodontists) before being adopted as instructional reference tools.

Finally, model performance can vary substantially. Künzle et al. [44] reported that ChatGPT-4 achieved 72% accuracy on endodontic assessment items, whereas GPT-3.5 performed as low as 25%. Such discrepancies underscore the need for task-specific validation and model selection before curricular adoption. Ongoing concerns about generative-AI hallucinations [45] further justify structured oversight—including faculty review of outputs, predefined guardrails for use during assessment, and clear guidance to students—to ensure that AI supports rather than undermines formative learning.

Across the five included studies, risk of bias was acceptable on QUADAS-2: three studies were low risk [20,22,23] and two were moderate risk [21,24], mainly due to participant-selection constraints, limited index-test reporting/threshold specification, and lack of external validation. Applicability concerns were low throughout.

Using GRADE for the comparative diagnostic outcome (AI vs. students), the certainty of evidence was judged moderate. We downgraded one level for inconsistency given substantial heterogeneity across tasks and model types (I^2^ ≈ 84% in the primary meta-analysis; I^2^ ≈ 77% in the sensitivity model). We did not downgrade for imprecision, because pooled CIs (and Bayesian CrIs) excluded the null; nor for risk of bias or indirectness, as studies used relevant student populations and endodontic tasks. Publication bias was not assessed (k < 10). Given the consistently large effects (SMD ≳ 1.0) favoring AI, we applied a small upgrade for magnitude, yielding an overall moderate certainty that AI outperforms students on the evaluated diagnostic tasks.

By contrast, the certainty for learner-centered educational outcomes (engagement, motivation, trust, accessibility) remains low to very low due to sparse data, heterogeneous constructs, and indirectness (no validated scales, no pre–post designs). The descriptive, unweighted differences were not graded and are provided only as contextual evidence consistent with the direction and approximate magnitude observed in the quantitative synthesis.

Part of the observed heterogeneity likely stems from architectural differences and task alignment. CNNs, which learn hierarchical spatial features, are well-suited to radiographic interpretation (e.g., periapical status detection) [46], whereas LLMs rely on language understanding and are sensitive to prompt design and stepwise reasoning requirements, which can alter stability and accuracy [47]. Multimodal/vision–language models that combine visual encoders with LLM backbones can bridge imaging and text but introduce additional variance due to connector design and fine-tuning data [48]. Consequently, architectural diversity not only contributes to statistical heterogeneity but also maps to different educational affordances (CNNs for visual feedback; LLMs for reflective reasoning). Explicit reporting of model family, version identifiers, and data provenance is therefore essential to interpretability and reproducibility [43].

A key strength of this review is its educational focus: eligibility was restricted to student-based studies, avoiding purely expert or bench-marking designs and thereby improving relevance to curriculum and assessment. Although working-length estimation was not classified as a diagnostic task, it was retained only in sensitivity and narrative analyses because of its educational relevance; this distinction does not alter the main diagnostic findings or their direction. In undergraduate endodontic training, working-length determination is a core competency and routine assessment target—explicitly expected in contemporary curriculum guidelines (radiographic and electronic methods) [49] and is frequently reported by students as a challenging step that requires structured training and feedback [50]. Moreover, evidence syntheses show that electronic apex locators often match or outperform radiographs [51], and WL accuracy can vary with operator experience [52], underscoring its nature as a measurable educational/assessment domain rather than a diagnostic construct. The work was reported under PRISMA 2020, applied QUADAS-2 at study level, and graded certainty with GRADE. Analytically, we used a conservative random-effects framework (Paule–Mandel with Hartung–Knapp adjustments) and complemented it with a Bayesian model using weakly informative priors, yielding convergent results and direct probability statements. Heterogeneity was handled transparently—pooling only accuracy when mean–SD data were available, conducting a sensitivity analysis with pre-specified SD imputation, and providing narrative synthesis for studies reporting only AUC/F1.

Although the inferences are encouraging, a few features of the evidence base invite measured interpretation. The dataset is modest, with two studies contributing to the primary meta-analysis and one additional study to a sensitivity model. This naturally limits precision and the scope for ancillary analyses (e.g., meta-regression or small-study bias tests). Between-study heterogeneity was notable (I^2^ ≈ 84% primary; 77% sensitivity), reflecting differences in tasks, model classes, settings, and student levels; the pooled estimates should therefore be read as average effects across diverse contexts.

Reporting and design features in several studies—such as missing SDs, single-center datasets, incomplete detail on index-test procedures/thresholds, and limited external validation for some CNN models—could influence observed performance. Learner-centered outcomes (engagement, motivation, trust, accessibility) were infrequently reported and typically lacked validated instruments or pre–post designs; accordingly, the certainty for these outcomes is low to very low, and conclusions about learning impact remain tentative. Publication bias was not evaluated (k < 10). Finally, results for rapidly evolving LLMs may change as models and prompts are updated; the Bayesian estimates presented are broadly consistent with the frequentist results, though exact probability values may vary under alternative reasonable priors.

Grounded in the moderate-certainty evidence that AI outperforms students on the diagnostic tasks evaluated here, the immediate educational value of these tools lies in augmentation rather than replacement. Large-language-model applications can act as formative tutors that scaffold case reasoning with explicit rationales and uncertainty statements, while image-based models can deliver rapid, criterion-referenced feedback in simulation and early clinical training (e.g., working-length estimation, radiographic interpretation). Used in co-scoring workflows with faculty oversight, AI can help standardize assessments and improve inter-rater consistency, provided that model/version control, prompt templates, and boundaries for formative versus summative use are defined in advance. Implementation should prioritize equity (course-level access rather than bring-your-own accounts), documentation of reliability and fairness, and faculty development focused on interpreting AI outputs, recognizing failure modes, and teaching explanation-first reasoning so that AI strengthens—rather than short-circuits—clinical judgment.

Finally, the findings of this review are consistent with recent systematic syntheses evaluating the diagnostic accuracy of artificial intelligence in endodontics and related dental fields [53,54,55]. The endodontics-focused synthesis confirms robust diagnostic performance of AI compared with clinicians [53], complementing our student-based perspective. Other contemporary reviews [54,55] similarly underscore AI’s diagnostic reliability and help situate the present results along a continuum from educational benchmarking to clinical validation—reinforcing that diagnostic accuracy, while not a direct learning endpoint, can serve as a quantifiable educational proxy when contextualized within structured endodontic training.

Future work should move beyond benchmarking to prospective, multicenter educational trials embedded in curricula, comparing usual instruction with instruction plus AI and measuring validated learner-centered outcomes alongside performance (OSCE/clinical scores, time-to-competency, retention, transfer to novel cases). A small core outcome set is needed to harmonize reporting across studies—covering learning, process (e.g., feedback cycles), reliability/fairness, student-reported measures (usability, trust, cognitive load), and safety—and trials should follow established guidance with transparent reporting of versions, prompts, thresholds, and drift monitoring. For imaging tasks, external validation on independent datasets across sites and devices is essential; for LLM tasks, locked versions and prompt protocols should be specified a priori. Finally, implementation of science and economic evaluations (feasibility, adoption, cost-effectiveness) and prospective evaluations of integrity/proctoring workflows will determine how AI can be integrated at scale in ways that are effective, fair, and sustainable.

## 5. Conclusions

This systematic review and meta-analysis focused on student-based evidence and found a consistent diagnostic advantage of AI over dental students in endodontic tasks. In the primary random-effects meta-analysis the pooled effect favored AI; a sensitivity model including one study with imputed SDs was similar. Bayesian analyses corroborated these findings. In absolute terms, the two LLM studies with analyzable mean–SD data showed about +20 percentage points higher accuracy for AI. These estimates represent average effects across heterogeneous tasks and models and should be interpreted accordingly.

Beyond comparative accuracy, educational outcomes remain sparsely measured. No study used validated instruments for engagement, motivation, trust, or accessibility, and no pre–post educational trials were identified; thus, certainty is moderate for the comparative diagnostic outcome but low to very low for learner-centered outcomes. Taken together, the current evidence indicates that AI consistently outperforms dental students on diagnostic tasks. While this suggests potential for AI to serve as a formative aid—providing objective and standardized feedback—such educational roles remain hypothetical and require validation through prospective, learner-centered studies using defined educational endpoints.

## Figures and Tables

**Table 1:** Summary of included studies and comparative performance of AI models and dental students across reported outcome metrics.

Study	AI Task	AI Model	Comparator (Student Level)	Outcome Metric	AI Metric(s) (%)	Student Metric(s) (%)
Durmazpinar et al. (2025) [20]	Endodontic case diagnosis	ChatGPT-4o (LLM)	3rd/5th year students	Accuracy (%)	91.4	60.8 (3rd-yr); 79.5 (5th-yr)
Basavanna et al. (2025) [21]	Working-length determination (in vitro; periapical X-rays)	Deep CNN	Postgraduate students	Accuracy (%)	85.0	75.4
Jin et al. (2024) [22]	C-shaped mandibular second molar detection (panoramic)	CNN (ResNet, DenseNet)	Graduate students (also reports novice dentists, specialists)	Accuracy (%), AUC	Accuracy 86.7% (ResNet-101, Group A); AUC 0.910 (95% CI 0.883–0.940)	Specialist = 81.2%; Novice dentist = 77.2%; Graduate student = 74.0%
Qutieshat et al. (2024) [23]	Endodontic condition diagnosis	ChatGPT-4 (LLM)	Junior and senior undergraduates	Accuracy (%)	99.0	77.0 (junior); 79.7 (senior)
Ramezanzade et al. (2023) [24]	Pulp exposure prediction	CNN + radiographic metrics	Dental students	F1-score, Accuracy, Sensitivity, Specificity, AUC	71.0, 78.0, 62.0, 83.0, 0.73	F1 = 58.0–61.0; Accuracy = 65.0–68.0

In this review, “endodontic case diagnosis” refers to identifying the pulpal and/or periapical diagnosis from comprehensive case vignettes combining symptoms, clinical test and radiographic findings, whereas “endodontic condition diagnosis” refers to classifying specific endodontic pathologies (e.g., reversible/irreversible pulpitis, pulp necrosis, apical periodontitis) using structured diagnostic assessments aligned with an expert gold standard.

**Table 2:** Exploratory differences in diagnostic accuracy between AI models and dental students.

Study	AI Accuracy (%)	Student Accuracy (%)	Difference (pp)
Durmazpinar et al. [20]	91.38	70.77 ^a^	+20.61
Basavanna et al. [21]	85.0	75.4	+9.6
Jin et al. [22]	86.70	74.00 ^b^	+12.7
Qutieshat et al. [23]	99.0	78.36 ^c^	+20.64
Ramezanzade et al. [24]	78.00	66.50 ^d^	+11.50
Unweighted mean (all 5)	not applicable	not applicable	+15.01
LLM subgroup mean	not applicable	not applicable	+20.63
CNN subgroup mean	not applicable	not applicable	+11.27
Sensitivity (excluding Qutieshat)	not applicable	not applicable	+13.60

pp = percentage points; CNN = Convolutional Neural Network; LLM = Large Language Model. ^a^ Durmazpinar [20]: combined student mean (3rd- and 5th-year; *n* = 70 and 80) using sample size weighting and standard formulas to average means and SDs; this value was used as the single student comparator. ^b^ Jin [22]: the comparator “students” was defined a priori as graduate students (74.0%); novice dentists and specialists were not considered students for this analysis. ^c^ Qutieshat [23]: weighted mean by *n* of junior (77.0%, *n* = 54) and senior (79.7%, *n* = 55) students → 78.36%. ^d^ Ramezanzade [24]: the midpoint of the reported accuracy range for students (65–68% → 66.5%) was used only for the unweighted descriptive summary; F1 metrics were not mixed with accuracy, and this study was not included in the quantitative meta-analyses.

**Table 3:** Risk of bias (QUADAS-2) for included diagnostic-accuracy studies.

Study	Participant Selection	Index Test (Conduct/Blinding; Prespecified Threshold)	Reference Standard	Flow & Timing	Overall
Durmazpinar 2025 [20]	Unclear	Low	Low	Low	Low
Basavanna 2025 [21]	Unclear	Unclear	Low	Low	Moderate
Jin 2024 [22]	Low	Low	Unclear	Low	Low
Qutieshat 2024 [23]	Unclear	Low	Low	Low	Low
Ramezanzade 2023 [24]	Unclear	Unclear	Unclear	Low	Moderate

Judgment levels follow QUADAS-2 domains: Patient selection; Index test; Reference standard; Flow & timing. Durmazpinar 2025 [20]: single-center student cohorts; item bank reviewed by endodontists (supports low risk for reference standard). Basavanna 2025 [21]: restricted radiographic dataset and no external validation; limited detail on algorithm thresholds (index test = unclear). Jin 2024 [22]: clear CNN–human comparison; ground-truthing procedure not fully specified in accessible text (reference standard = unclear). Qutieshat 2024 [23]: standardized cases with expert-defined gold standard and student cohorts; low flow/timing concerns. Ramezanzade 2023 [24]: retrospective case selection with intended class imbalance; limited masking/reporting details for index test and reference standard. Overall risk: Low if all domains low/unclear with no high-risk domain; Moderate when ≥1 domain high or multiple domains unclear.

**Table 4:** Summary of Findings (GRADE Assessment).

Outcome	Studies Contributing	Effect Estimate (SMD, 95% CI)	Certainty (GRADE)	Footnotes
Diagnostic accuracy (AI vs. students)—primary meta-analysis	k = 2 (studies [20,23])	Hedges g = 1.48 (95% CI 0.60–2.36)	Moderate (⨁⨁⨁◯)	Downgraded for inconsistency (I^2^ ≈ 84%) ^a^; not downgraded for imprecision ^b^; no serious risk-of-bias or indirectness concerns ^c^; publication bias not assessed (k < 10).
Diagnostic accuracy (AI vs. students)—sensitivity (incl. SD imputation)	k = 3 (adds [21])	Hedges g = 1.45 (95% CI 0.77–2.14)	Moderate (⨁⨁⨁◯)	Same judgment; I^2^ ≈ 77%. Large and consistent direction of effect across all five student-based studies supports upgrading one level for magnitude ^d^.

^a^ Inconsistency: heterogeneity substantial (I^2^ ≈ 84% primary; 77% sensitivity) due to variation in tasks/models; direction of effect consistent. ^b^ Imprecision: CIs are wide but exclude the null; Bayesian CrIs also exclude 0, supporting precision adequate for decision-making at this stage. ^c^ Risk of bias/indirectness: three low-risk and two moderate-risk studies; tasks and populations align with endodontic education; no downgrade overall. ^d^ Upgrade for large effect: pooled SMD ≳ 1.0 with consistent superiority of AI across all five studies (including narrative results). Publication bias: not evaluated because k < 10; no downgrade. ⨁⨁⨁◯ indicates moderate certainty of evidence based on the GRADE approach. The GRADE system uses four levels (high, moderate, low, very low) to evaluate the confidence in effect estimates across studies. SMD: Standardized Mean Difference, used to compare effect sizes across studies with different outcome scales.

## Data Availability

The original contributions presented in this study are included in the article. Further inquiries can be directed to the corresponding author.

## Supplementary Materials

The following supporting information can be downloaded at: https://www.mdpi.com/article/10.3390/dj13110489/s1, Table S1. PRISMA 2020 checklist; Table S2. Complete search strategies for all databases; Table S3. Studies excluded after full-text screening and reasons for exclusion.
