# Supplementary material for: Artificial Intelligence in Endodontic Education: A Systematic Review with Frequentist and Bayesian Meta-Analysis of Student-Based Evidence
Source: Dent J (Basel). 2025 Oct 23;13(11):489. doi: 10.3390/dj13110489 (PMC12650973; doi:10.3390/dj13110489)
Supplement: Supplementary file 1 [file dentistry-13-00489-s001.zip › Supplementary Table S2. Search Strategies.pdf]

Supplementary Table S2. Complete search strategies for all databases

Database	Search Strategy	Time Window
PubMed/MEDLINE	((("artificial intelligence"[MeSH Terms] OR "machine learning"[MeSH Terms] OR "deep learning"[All Fields] OR "chatbot"[All Fields] OR "large language model"[All Fields]) OR ("artificial intelligence"[All Fields] OR "machine learning"[All Fields] OR "deep learning"[All Fields] OR "chatbot"[All Fields] OR "large language model"[All Fields])) AND ("endodontics"[MeSH Terms] OR "endodontics"[All Fields]) AND ("students"[MeSH Terms] OR "dental students"[All Fields] OR "education"[MeSH Terms] OR "training"[All Fields] OR "assessment"[All Fields]))	Inception – July 2025
Embase	('artificial intelligence'/exp OR 'machine learning'/exp OR 'deep learning' OR 'chatbot' OR 'large language model') AND ('endodontics'/exp OR endodontics) AND ('student'/exp OR 'dental student' OR 'education'/exp OR training OR assessment)	Inception – July 2025
Scopus	TITLE-ABS-KEY ("artificial intelligence" OR "machine learning" OR "deep learning" OR "chatbot" OR "large language model") AND TITLE-ABS-KEY ("endodontics") AND TITLE-ABS-KEY ("students" OR "dental students" OR "education" OR "training" OR "assessment")	Inception – July 2025
Web of Science Core Collection	TS=("artificial intelligence" OR "machine learning" OR "deep learning" OR "chatbot" OR "large language model") AND TS=("endodontics") AND TS=("students" OR "dental students" OR "education" OR "training" OR "assessment")	Inception – July 2025
Google Scholar	"artificial intelligence" OR "machine learning" OR "deep learning" OR "chatbot" OR "large language model" AND endodontics AND ("students" OR "dental students" OR "education" OR "training" OR "assessment")	Inception – July 2025
