# Supplementary material for: Artificial Intelligence in Endodontic Education: A Systematic Review with Frequentist and Bayesian Meta-Analysis of Student-Based Evidence
Source: Dent J (Basel). 2025 Oct 23;13(11):489. doi: 10.3390/dj13110489 (PMC12650973; doi:10.3390/dj13110489)
Supplement: Supplementary file 1 [file dentistry-13-00489-s001.zip › Supplementary Table S3. Studies excluded after full-text screening and reasons for exclusion.pdf]

Supplementary Table S3. Studies excluded after full-text screening and reasons for exclusion

Study	Reason for Exclusion
Karkehabadi H, Khoshbin E, Ghasemi N, Mahavi A, Mohammad-Rahimi H, Sadr S. Deep learning for determining the difficulty of endodontic treatment: a pilot study. BMC Oral Health. 2024 Dec;24(1):574. doi:10.1186/s12903-024-04235-4.	No student involvement; educational utility only discussed in future-oriented statements
Çelik B, Genç MZ, Çelik ME. Evaluation of root canal filling length on periapical radiograph using artificial intelligence. Oral Radiol. 2025 Jan;41(1):102–110. doi:10.1007/s11282-024-00781-3.	Used patient-based clinical data without integration into student-generated framework
Gowdar IM, Alateeq AA, Alnawfal AMA, Alharbi AFA, Alhabshan AMS, Aldawsari SMS, AlHarbi NAH. Artificial Intelligence and its Awareness and Utilization among Dental Students and Private Dental Practitioners at Alkharj, Saudi Arabia. J Pharm Bioallied Sci. 2024 Jul;16(Suppl 3):S2264-S2267. doi: 10.4103/jpbs.jpbs_188_24.	Non-endodontic focus without separate endodontic outcomes; absence of direct performance comparison between AI and students or experts; and lack of primary diagnostic accuracy metrics.
Sarhan S, Badran A, Ghalwash D, Gamal Almalahy H, Abou-Bakr A. Perception, usage, and concerns of artificial intelligence applications among postgraduate dental students: cross-sectional study. BMC Med Educ. 2025 Jun 23;25(1):856. doi: 10.1186/s12909-025-07544-6.	Although the study included postgraduate dental students, it focused exclusively on evaluating AI performance in a clinical setting without integrating the tool into an educational or assessment framework, and it did not compare AI results with student-generated outputs or validated educational benchmarks.
Kazimierczak W, Kazimierczak N, Issa J, Wajer R, Wajer A, Kalka S, Serafin Z. Endodontic treatment outcomes in cone beam computed tomography images—assessment of the diagnostic accuracy of AI. J Clin Med. 2024;13(14):4116. doi:10.3390/jcm13144116.	The study did not focus on endodontics but instead addressed oral radiology training more broadly. Although it involved dental students, the AI application was not directly integrated into endodontic education, assessment, or diagnostic training. Furthermore, no outcomes specific to

	endodontic tasks were reported, and AI performance was not benchmarked against student results in an endodontic context. These factors render the study outside the defined scope of this review.
Hasan HA, Saad FH, Ahmed S, Mohammed N, Farook TH, Dudley J. Experimental validation of computer-vision methods for the successful detection of endodontic treatment obturation and progression from noisy radiographs. Oral Radiol. 2023;39(4):683–98. doi:10.1007/s11282-023-00850-2.	This study was excluded because it did not meet the comparator criterion defined in our review. Although it assessed AI-based diagnostic support in dentistry, the evaluation was performed against expert clinician standards only, with no direct involvement of dental students or benchmarking against student-generated outputs. Additionally, the AI tasks evaluated were not integrated within an endodontic educational or assessment framework.
Li Y, Zeng G, Zhang Y, et al. AGMB-Transformer: anatomy-guided multi-branch Transformer network for automated evaluation of root canal therapy. IEEE J Biomed Health Inform. 2022;26(4):1684–95. doi:10.1109/JBHI.2021.3123456.	This study was excluded because it did not involve dental students at any stage of formal endodontic training, nor did it benchmark AI performance against student-generated outputs or validated student assessment instruments. The evaluation compared AI solely to baseline model performance for automated root canal therapy assessment on radiographs, with no educational or student-focused integration.
Szabó V, Orhan K, Dobó-Nagy C, Veres DS, Manulis D, Ezhov M, Sanders A, Szabó BT. Deep Learning-Based Periapical Lesion Detection on Panoramic Radiographs. Diagnostics (Basel). 2025 Feb 19;15(4):510. doi: 10.3390/diagnostics15040510.	Lack of student involvement, absence of an educational setting, and no comparator against student performance led to its exclusion.
Fan W, Zhang J, Wang N, Li J, Hu L. The Application of Deep Learning on CBCT in Dentistry. Diagnostics (Basel). 2023 Jun 14;13(12):2056. doi: 10.3390/diagnostics13122056.	The study was excluded because it did not involve dental students at any stage of training, lacked integration into an educational or assessment framework, and did not benchmark AI performance against student results or validated student assessment instruments, instead comparing

	solely to expert or dataset-based ground truths.
Pauwels R, Brasil DM, Yamasaki MC, Jacobs R, Bosmans H, Freitas DQ, Haiter-Neto F. Artificial intelligence for detection of periapical lesions on intraoral radiographs: Comparison between convolutional neural networks and human observers. Oral Surg Oral Med Oral Pathol Oral Radiol. 2021 May;131(5):610-616. doi: 10.1016/j.oooo.2021.01.018.	No involvement of students or educational benchmarks
Abdelazim R, Fouad EM. Artificial intelligent-driven decision-making for automating root fracture detection in periapical radiographs. BDJ Open. 2024 Oct 1;10(1):76. doi: 10.1038/s41405-024-00260-1.	Although it involves dental radiographs and AI-based analysis, it does not include participation of dental students at any stage of training, nor does it benchmark AI performance against student results obtained within an educational or assessment framework. The AI evaluation is conducted in a purely clinical context, focusing on patient cases, and there is no integration into an educational setting or use of validated student assessment instruments.
Çatmabacak ED, Çetinkaya İ. Deep learning algorithms for detecting fractured instruments in root canals. BMC Oral Health. 2025 Feb 23;25(1):293. doi: 10.1186/s12903-025-05652-9.	The study was excluded because, despite employing an AI model to evaluate radiographic features in root canal therapy, it did not involve dental students or any student-generated data, nor did it integrate the AI tool within an educational or assessment framework relevant to endodontic training. The study population consisted entirely of clinical patient cases, and no benchmarking against student or expert performance within an educational context was reported.
Yadalam PK, Anegundi RV, Ardila CM. Evolution Oroinformatics: A Deep Learning Perspective in Personalised Dental Care. Int	It is a Letter to the Editor, not an original research study, and it does not involve empirical evaluation of chatbot-assisted learning or any other educational intervention with real dental students. It is a

Dent J. 2024 Oct;74(5):1174-1175. doi: 10.1016/j.identj.2024.05.009.	conceptual/opinion piece discussing the integration of evolutionary bioinformatics and deep learning in dentistry, without presenting primary data and study participants, outcomes.
Hadzic A, Urschler M, Press JA, Riedl R, Rugani P, Štern D, Kirnbauer B. Evaluating a Periapical Lesion Detection CNN on a Clinically Representative CBCT Dataset-A Validation Study. J Clin Med. 2023 Dec 29;13(1):197. doi: 10.3390/jcm13010197.	Although it explores AI-assisted analysis in dentistry, it does not directly involve dental students or use validated student assessment instruments in endodontic education. The dataset and evaluation focus are entirely clinical, with no educational or training component
Orhan K, Bayrakdar IS, Ezhov M, Kravtsov A, Özyürek T. Evaluation of artificial intelligence for detecting periapical pathosis on cone-beam computed tomography scans. Int Endod J. 2020 May;53(5):680-689. doi: 10.1111/iej.13265.	It does not involve dental students or any educational context relevant to endodontic training. The research focuses exclusively on clinical CBCT datasets from patients, evaluating AI performance against manual expert segmentation, without student participation, benchmarking with student results, or use of validated student assessment instruments.
Jin L, Du B, Xu Z, Bai H, Ding P, Zhang Z, Pan Y, Lin Y, Li Z, Rausch-Fan X, Hu F, Zhang X. Deep-learning network for automated evaluation of root-canal filling radiographic quality. Eur J Med Res. 2025 Apr 17;30(1):297. doi: 10.1186/s40001-025-02331-x.	Although it involved AI-based detection of anatomical variations relevant to endodontics (C-shaped canals), it was conducted entirely with patient CBCT data and lacked direct involvement of dental students or integration into an educational or assessment framework.
Yang P, Guo X, Mu C, Qi S, Li G. Detection of vertical root fractures by cone-beam computed tomography based on deep learning. Dentomaxillofac Radiol. 2023 Feb;52(3):20220345. doi: 10.1259/dmfr.20220345.	Despite applying deep learning for endodontic image analysis, it did not involve dental students, student-generated data, or any validated educational assessment framework. The dataset was entirely clinical, and the AI model's performance was benchmarked against expert examiners only, without any comparison to student outcomes or integration into an educational context
Chen YW, Stanley K, Att W. Artificial intelligence in dentistry: current applications	It is a narrative review providing a general overview of artificial intelligence applications

and future perspectives. Quintessence Int. 2020;51(3):248-257. doi: 10.3290/j.qi.a43952. Erratum in: Quintessence Int. 2020;51(5):430. doi: 10.3290/j.qi.a44465.	in dentistry without focusing on endodontics or involving any original data collection, student participation, or comparative evaluation between AI and students.
Liu J, Liu X, Shao Y, Gao Y, Pan K, Jin C, Ji H, Du Y, Yu X. Periapical lesion detection in periapical radiographs using the latest convolutional neural network ConvNeXt and its integrated models. Sci Rep. 2024 Oct 25;14(1):25429. doi: 10.1038/s41598-024-75748-9.	It is a narrative review describing applications of artificial intelligence across various dental specialties, including endodontics, but it does not present original experimental or observational data involving dental students, nor does it benchmark AI performance against student outcomes.
Li CW, Lin SY, Chou HS, Chen TY, Chen YA, Liu SY, Liu YL, Chen CA, Huang YC, Chen SL, Mao YC, Abu PAR, Chiang WY, Lo WS. Detection of Dental Apical Lesions Using CNNs on Periapical Radiograph. Sensors (Basel). 2021 Oct 24;21(21):7049. doi: 10.3390/s21217049.	Although it focuses on automated evaluation of dental radiographs using AI, it does not involve dental students or any benchmarking of AI performance against student results. The study is purely a technical performance evaluation of the proposed model, with no educational or comparative student component, making it ineligible under your inclusion criteria.
Ba-Hattab, R.; Barhom, N.; Osman, S.A.A.; Naceur, I.; Odeh, A.;Asad, A.; Al-Najdi, S.A.R.N.; Ameri,E.; Daer, A.; Silva, R.L.B.D.; et al. Detection of Periapical Lesions on Panoramic Radiographs Using Deep Learning. Appl. Sci. 2023, 13, 1516.https://doi.org/10.3390/app13031516	Although it involved dental students, it was a perception-based cross-sectional survey assessing attitudes toward AI integration in dentistry rather than an evaluation of AI model performance in endodontics. The study lacked an endodontic focus, did not report any AI performance metrics (e.g., accuracy, sensitivity, specificity, AUC), and did not include benchmarking of AI outcomes against student or expert clinical performance, making it incompatible with the predefined eligibility criteria.
Santos-Junior AO, Fontenele RC, Neves FS, Tanomaru-Filho M, Jacobs R. A novel artificial intelligence-powered tool for automated root canal segmentation in single-rooted teeth on cone-beam computed	The participant pool consisted of image datasets without direct involvement of dental students, and no comparison was made against student performance in an educational or assessment setting. The study's primary focus was algorithmic detection and validation, not the evaluation of AI within an

tomography. Int Endod J. 2025 Apr;58(4):658-671. doi: 10.1111/iej.14200.	endodontic educational framework or benchmarking against student outcomes.
Wang K, Zhang S, Wei Z, Fang X, Liu F, Han M, Du M. Deep learning-based efficient diagnosis of periapical diseases using dental X-rays. Image Vis Comput. 2024;147:105061. doi:10.1016/j.imavis.2024.105061	Although it involves AI applied to dental imaging, it does not feature direct participation of dental students, nor does it benchmark AI performance against student results within an educational or assessment context. Instead, the study focuses on automated radiographic analysis for clinical diagnosis, without any student-related outcomes or educational comparators, making it ineligible for inclusion.
Ver Berne J, Saadi SB, Oliveira-Santos N, Marinho-Vieira LE, Jacobs R. Automated classification of panoramic radiographs with inflammatory periapical lesions using a CNN-LSTM architecture. J Dent. 2025 May;156:105688. doi: 10.1016/j.jdent.2025.105688.	Although it involves AI-assisted image analysis in a dental context, it does not include participation or outcome measurement involving dental students, nor does it benchmark AI performance against student results within an educational or assessment framework. Additionally, its focus is on general radiographic analysis and image segmentation rather than specifically addressing endodontic education or training outcomes
Liu J, Jin C, Wang X, Pan K, Li Z, Yi X, Shao Y, Sun X, Yu X. A comparative analysis of deep learning models for assisting in the diagnosis of periapical lesions in periapical radiographs. BMC Oral Health. 2025 May 26;25(1):801. doi: 10.1186/s12903-025-06104-0.	This study focuses on the development and evaluation of a deep learning model for automated tooth numbering on panoramic radiographs, without involving dental students or benchmarking AI performance against student results in an educational or assessment context.
Viet DH, Son LH, Tuyen DN, Tuan TM, Thang NP, Ngoc VTN. Comparing the accuracy of two machine learning models in detection and classification of periapical lesions using periapical radiographs. Oral Radiol. 2024 Oct;40(4):493-500. doi: 10.1007/s11282-024-00759-1.	This study evaluates the performance of two deep learning models (Faster R-CNN and YOLOv4) for detecting and classifying periapical lesions using periapical radiographs, with diagnoses from experienced dentists as the reference standard. It does not involve dental students, nor does it benchmark AI performance

	against student results or outcomes in an educational or assessment framework.
Moidu NP, Sharma S, Chawla A, Kumar V, Logani A. Deep learning for categorization of endodontic lesion based on radiographic periapical index scoring system. Clin Oral Investig. 2022 Jan;26(1):651-658. doi: 10.1007/s00784-021-04043-y.	This study focuses exclusively on developing and testing a convolutional neural network (YOLOv3) for automated PAI scoring of periapical lesions on intraoral periapical radiographs. It does not involve dental students or compare AI performance with student diagnostic accuracy. Additionally, the work is aimed at clinical automation rather than educational assessment, further disqualifying it from inclusion.
Prinz M, Schäfer E, Bürklein S, Donnermeyer D. Endodontic diagnostics training in undergraduate dental education: An observational pilot study on AI-driven virtual patient e-learning. Int Endod J. 2025 Jul 22. doi: 10.1111/iej.14277.	Observational pilot based on self-perception (Likert questionnaires) without validated educational outcomes or objective performance metrics; no benchmarking of AI versus students within a standardized evaluation framework.
Ibrahim M, Omidi M, Guentsch A, Gaffney J, Talley J. Ensuring integrity in dental education: Developing a novel AI model for consistent and traceable image analysis in preclinical endodontic procedures. Int Endod J. 2025 Jun 19. doi: 10.1111/iej.14273.	Development/validation of a Siamese network model for integrity control of radiographs; does not evaluate educational outcomes or compare AI with students; no integration into a validated teaching/assessment framework.
Ayhan M, Kayadibi İ, Aykanat B. RCFLA-YOLO: a deep learning-driven framework for the automated assessment of root canal filling quality in periapical radiographs. BMC Med Educ. 2025 Jul 1;25(1):894. doi: 10.1186/s12909-025-07483-2.	Technical AI study on radiographs from preclinical exercises, but without direct student participation in learning evaluation, no student comparator, and no use of validated educational instruments; focus is solely on algorithmic performance.
Künzle P, Paris S. Performance of large language artificial intelligence models on solving restorative dentistry and endodontics student assessments. Clin Oral Investig. 2024 Oct 7;28(11):575. doi: 10.1007/s00784-024-05968-w.	Comparison of LLMs on question banks covering restorative dentistry and endodontics, without student participation or student outcomes; endodontic results are not reported separately and no student comparator provided; outside the defined scope.
